# Dental Problems and Prophylactic Care in Cats—Knowledge and Perceptions among Swedish Cat Owners and Communication by Veterinary Care Staff

**DOI:** 10.3390/ani11092571

**Published:** 2021-08-31

**Authors:** Kristin Oskarsson, Louise Axelsson Puurtinen, Johanna Christina Penell

**Affiliations:** 1 Evidensia Specialist Animal Hospital Strömsholm, Djursjukhusvägen 11, 734 94 Strömsholm, Sweden; Kristin.Oscarsson@evidensia.se; 2 AniCura Gärdets Animal Clinic, Sandhamnsgatan 65, 115 28 Stockholm, Sweden; Louise.axelsson.Puurtinen@anicura.se; 3 Department of Clinical Sciences, Swedish University of Agricultural Sciences, 750 07 Uppsala, Sweden

**Keywords:** cat, oral health, dental, knowledge, perceptions, prevention, survey, veterinarian, veterinary nurse, owner

## Abstract

**Simple Summary:**

Dental disease has a negative impact on the quality of life and welfare of various species. The most important preventive measures in cats are performed by the cat owners. However, cat owners’ knowledge of dental disease and prevention is unclear. Veterinary care staff are important sources of information to the cat owners, but the type and frequency of information on dental disease prevention given to the owners have not been described. We conducted web-based surveys to investigate owners’ knowledge of and veterinary care staff’s communication of dental problems in cats. Overall, 2/3 of the cat owners knew about dental disease and listed modified eating behaviour, gingivitis, halitosis, pain and dental calculus as the most common signs. Commonly, the source of information was the internet followed by veterinary care staff, and the most known preventive measure was tooth brushing, although a small proportion performed it daily or every second day. Veterinary care staff responded that they always or sometimes informed cat owners about prophylactic dental care, most frequently by oral communication, and tooth brushing was the most common preventive measure recommended. This study indicates that cat owners have the relevant knowledge to support the oral health of cats, but the application of preventive measures needs to increase to promote welfare in cats. In particular, the motivation and practical options for owners of cats who may not tolerate tooth brushing should be considered, and alternative strategies discussed. Veterinary care staff present relevant communication but there is room to develop strategies such as improvement in education and communication.

**Abstract:**

Dental problems are increasingly recognised in cats although many problems are preventable by tooth brushing. However, the knowledge level and preventive measures performed by owners are unclear. Additionally, there is a shortage of information on the communication by veterinary care staff to owners on dental health and prophylaxis in cats. The aim was to describe the knowledge and perceptions among Swedish cat owners and the communication by veterinary care staff on dental problems and prevention in cats. We distributed two electronic surveys; to cat owners and care staff, respectively. Of the cat owners, *n* = 407, 2/3 stated that they have knowledge about dental disease, listing modified eating behaviour as the most common sign followed by gingivitis, halitosis, pain and dental calculus. The main source of information was the internet followed by veterinary care staff, and 47% of the owners reported that they perform preventive oral health measures. The single most frequently stated preventive measure was tooth brushing, which was applied by 28% of the cat owners but with low frequency (daily *n* = 15, every second day *n* = 14). Veterinary care staff, *n* = 179, reported that they inform (47%) or sometimes inform (42%) cat owners on prophylactic dental care, daily or 3–7 times a week (combined 39%) most frequently by oral communication, with tooth brushing as the most common measure recommended. This study suggests that cat owners have relevant knowledge on dental health in cats, but the performed preventive measures are too infrequent to support good oral health in cats. There is room to develop strategies such as improvement in education and communication to increase welfare in cats. This includes consideration of the motivation and practical options for owners of cats who may not tolerate tooth brushing.

## 1. Introduction

Dental problems in cats are increasingly recognised globally and the body of evidence regarding the relationship between oral health, general health and longevity in several species including humans, dogs and cats is growing [1,2,3]. Relationships between periodontal disease in cats and the development of cardiorespiratory, hepatic, and renal disorders have been suggested [4]. Oral disease has been shown to have a negative impact on quality of life and welfare in various species, and the relief of oral pain and suffering for cats has been claimed to be within reach for the veterinary practitioner [5]. It was suggested that because the human dental profession has educated clients well, it has become easier for animal owners to understand the need for adequate dental care also for their animals [5]. However, there is a lack of information on cat owners’ knowledge regarding dental health and prevention in cats.

Dental problems are common in cats. For example, periodontal disease was the single most prevalent diagnosis-level disorder in cats based on clinic data in the UK [6]. Furthermore, a survey among pure breed cat owners in Finland reported that dental and oral diseases were the most prevalent disease category in cats (prevalence 28%), and dental calculus and gingivitis were the most prevalent clinical signs reported both in all cats and in most of the breeds [7]. The important first step in the assessment of oral health status is owner observation for dental issues and regular veterinary visits. In addition to the oral examination, full-mouth radiography by a veterinarian was recommended [8]. A variety of dental problems can be reversed and controlled with proper treatment and adequate dental care at home [9]. The combination of veterinary examination and professional dental cleaning, together with daily dental home care, is the foundation for good dental health [10]. To reduce the occurrence of dental problems, it is of crucial importance that cat owners have adequate knowledge about dental diseases and problems. There is supportive evidence that dog owners are able to perform a relative assessment of their dog’s dental health status [11]. Although the attitudes, knowledge and care practices of cat owners regarding cat behavior and environmental needs of cats have been investigated [12], it is unclear what type and frequency of preventive measures the owners apply on their cats and where they learned about dental care.

In humans, mechanical cleaning with a toothbrush is one of the most common methods used to remove plaque [13]. Toothbrushing has become an increasingly common prevention method also in pets and daily tooth brushing is considered the gold standard for preventing dental problems in pets [14,15]. In dogs, daily or every other day toothbrushing was proven to be a sufficient prophylactic measure [16]. However, studies on tooth brushing and other prophylactic measures in cats are scarce regarding the efficacy and recommended frequency, and owner knowledge about this preventive measure. Furthermore, the role of the veterinary care staff in communicating information on oral health and prophylaxis to cat owners is unclear.

The aim of this study was to investigate the knowledge among Swedish cat owners on prophylactic dental care and dental health in cats as well as to describe the information intermediation from animal health care personnel to cat owners regarding dental health and prevention.

## 2. Materials and Methods

### 2.1. Study Design

The data collection was part of a bachelor thesis project at the veterinary nursing program at the Swedish University of Agricultural Sciences (SLU). Two anonymous online surveys, in Swedish, were developed by the first two authors in the web program Netigate; one cat owner survey (COS), and one veterinary care staff survey (VCS). The surveys were validated in two steps before distribution to the intended recipients: (i) by veterinary nursing students, (ii) a test group consisting of fourteen persons providing constructive criticism on the content and question flow. The surveys were revised based on these comments before they were released. The data from the test group were not included in the analysis. For COS, the target group was cat owners in Sweden and the sample frame was membership in certain cat groups on the social media platform Facebook. The survey was posted in six different groups on Facebook after approval of the groups’ administrator and included both general groups (i.e., for all breeds, *n* = 2) and groups for a certain cat breed (*n* = 4). For VCS, the target was veterinary care staff in Sweden and the sample frame included small animal clinics and hospitals in the SLU network of clinical partners for extramural training for veterinary nurse students. Both surveys included a section where the approval of the respondents of data collection and analysis was mandatory to continue with the survey. The two surveys were both anonymous, and therefore no personal identification information was collected about the respondents. The surveys contained a maximum of fourteen questions but depending on how the respondents answered, the number of questions answered could vary. All questions in the survey were mandatory, with the exception of the two last questions which were optional, where the respondents could leave comments on the surveys. The surveys included multiple-choice questions, with options to enter additional information on some questions, as well as single choice questions where the respondent could choose one response from a set of given alternatives, and a few open-ended questions.

To participate in Survey 1 (COS), it was required that the respondent owned a cat. The survey was divided into five sections. The first section collected information about the respondent and their cat, such as the breed of the cat and previous treatment of dental problems. The second section collected information about the cat owner’s knowledge of dental problems and signs, and how this information was received, as well as the occurrence of prophylactic dental care. The third and fourth sections were only available to answer if the respondents performed any prophylactic dental care on their cats. The questions regarded which kind of prophylactic dental care they performed, and if they brushed their cat’s teeth, and, if so, the frequency of tooth brushing. The fifth and last section contained questions regarding the respondent’s opinion on how the information transmission from the animal health care personnel has been. The survey was available from 8 to 15 February 2020.

Survey 2 (VCS) was shared by online correspondence with a total of 44 different veterinary clinics and hospitals. Emails were sent out, with a short introduction text explaining the purpose of the study as well as a link to the survey. In order to participate in the survey, it was required that the respondent was actively working in one of the following professions: veterinarian, veterinary nurse, or animal caretaker. The survey contained five sections. The first section collected information about the respondent and their workplace, such as the size of the workplace and the respondent’s experience in the profession and with feline dental care. The second section included questions about the workplace, the number of dental patients that visit the workplace and if the respondent informs cat owners about prophylactic dental care. Sections three and four were only available for the respondents who stated that they give some kind of information about prophylactic dental care. In section three, the questions were about how, and in which context, they inform cat owners about dental care, the frequency of information about dental care, and the type of prophylactic measures they recommend to cat owners. Section four contained questions regarding which dental problems and signs the respondent give information about and if they are experiencing that the cat owners understand them about the importance of prophylactic dental care in cats. The fifth and last section contained an optional question where the respondent could leave comments on the survey. The survey was available from 10 February to 24 February 2020.

### 2.2. Data Analysis

Surveys that were not completed were excluded from the analysis. Owners were assigned to only own the breed they listed, for one or more cats, when reporting one breed regardless if the cats were specified purebred or housecat. Owners that reported two different breeds were classified to own purebred cats if at least one of the breeds were purebred, otherwise categorised as a house cat owner. The question regarding knowledge of clinical signs of dental problems was either yes or no, where the yes response provided the option of specifying in free text examples of clinical signs. The free text answers were analysed and categorised into the following groups: modified eating behavior, gingivitis, halitosis, pain, dental calculus, Feline Resorptive Lesions (FORL), tooth loss, sialorrhea and weight loss. Modified eating behavior includes for example signs of inappetence, dropping food whilst eating, refusing dry food and dry treats, and difficulty chewing. For veterinary care staff, they were either based at a veterinary clinic or a veterinary hospital. A veterinary clinic was defined as “a smaller business that often is more specialised, e.g., small animals or horses, and “most often do not have stationary care facilities so that animals in need of medical ward treatment will be referred to a veterinary hospital. Clinics do not provide off hours service”. Profession was categorised as a veterinary surgeon (including fully licenced and temporarily licenced awaiting final educational credits), veterinary nurse (including fully licenced and temporarily licenced awaiting final educational credits) or animal caretaker (with no specified education).

### 2.3. Statistical Methods

Descriptive statistics were calculated. Proportions are presented with 95% confidence intervals. Comparison between proportions was made using Pearson’s chi-squared test, except when cell numbers were low and Fisher’s exact test was used instead. The level for statistical significance was set to *p* = 0.05, however, when multiple tests were performed this limit for statistical significance was adjusted using the Bonferroni correction, i.e., dividing the *p*-value for significance with the number of tests performed, to lower the alpha value and avoid spurious positives. Data were transferred from Netigate to Excel and then into Stata format. All analyses were performed using Stata SE 16.1 (StataCorp, College Station, TX, USA).

## 3. Results

### 3.1. Cat Owner Survey

In total, 462 cat owners started filling out the survey and 407 finished. The results are based on the completed surveys. The median time for filling out the entire questionnaire was 3 min (range from <1 min to 556 min with three observations > 40 min; 556, 113 and 86 min, respectively, 95% confidence interval 3.8–8.5 min).

Table 1 describes the cat owners’ responses and characteristics. Of the respondents, 228 (56%) had housecats and 179 (44%) had purebred cats, either one breed (*n* = 173), two different breeds (*n* = 4) or pure breed and housecat (*n* = 2). The owners reportedly owned 26 different breeds of cats, in addition to the house cat. The most common breeds were Cornish Rex and Devon Rex (37 respondents each), followed by Persian (*n* = 17), Ragdoll and Burmese (13 respondents each). Of the house cat respondents, 59% reported that their cat was indoors whereas the corresponding frequency among purebred cat respondents was 91% (statistically significant difference; *p* < 0.001). Membership in breed club was 1% and 40% for housecats and purebred cats, respectively. Thirty-eight respondents were cat breeders, 46 had experience from participating in cat shows and 57 worked currently or previously as veterinary care staff. In total, 112 (28%) reported that they did not know of any clinical signs indicating dental problems, whereas 295 (72%) listed signs they knew to be related to dental problems in cats. The cat owners’ free-text descriptions of known signs were grouped into nine categories which were aggregated into three areas; weight loss, pain, and related to the mouth and oral cavity, respectively, and are presented in Table 2. For the 286 owners who reported signs, the number of signs reported by the owner ranged from 1 to 7 with 85% reporting 1–3 and 95% reporting 1–4 signs. For the subgroup of cats that had previous dental problems (*n* = 112, see Table 1), 102 owners reported known signs of dental problems. The most frequently known clinical sign was gingivitis (52%) followed by modified eating behavior (51%), halitosis (37%), pain (29%), FORL (20%), dental calculus (18%), Sialorrhea (11%), tooth loss (3%), and weight loss (2%).

#### 3.1.1. Sources of Information on Dental Problems in Cats

Overall, 54 persons reported that they had not received any information on dental problems. The other respondents (*n* = 353) stated that they had received information from one or more of the following sources: veterinary care staff, internet, books or journals, friend, breeder, course/education, do not remember where from, and other, see Table 3. Of the 26 replies for “other”, 15 reported information from multiple sources, 7 from personal experience and 4 by professional experience in related professions. The 26 responses included sources of information such as through own experience (*n* = 11), through online groups including Facebook (*n* = 6), and sources such as insurance companies and breeding club seminars (*n* = 9). Table 3 also includes the information on how the owners experienced the information from veterinary sources.

Three hundred and seventy-five respondents reported knowledge on the following problems (more than one problem could be registered: dental calculus (*n* = 345), FORL (*n* = 309), gingivitis (*n* = 309), tooth loss (*n* = 214), fractured tooth (*n*= 201), and malocclusion (*n* = 168). There was no statistical difference between owners of purebred cats or house cat owners except for knowledge on FORL (*p* < 0.001) where a higher proportion of purebred cat owners knew about this problem.

#### 3.1.2. Preventive Measures by Cat Owners

In total, 192 (47%) cat owners stated that they performed any preventive oral health measures, and 167 of them provided details on the types and frequency of preventive measures they apply, see Table 4. Of the 17 free-text responses describing other options than listed in Table 4, 10 reported visits to a veterinarian or nurse to check the dental status, and 4 reported that they gave their cats various bones to chew on. Of the owners reporting to use dental treats or dental food, 15 claimed to use both. The owners reporting tooth brushing (*n* = 113) claimed to brush daily (*n* = 15), every second day (*n* = 14), every third day (*n* = 18), once per week (*n* = 32), once per month (*n* = 18) and more seldom (*n* = 16). A significantly higher proportion of the owners claiming that tooth brushing is the preventive measure they perform described the information from clinics and hospitals as very good or good (*p* = 0.02) compared to owners that did not perform tooth brushing on their cat.

For the subgroup of cat owners that reported previous dental problems in their cat, 48 reported on the preventive measures they take, in total 74 preventive actions. The most common action was tooth brushing 80%), followed by a dental treat (23%), dental food (21%), dental toy (13%) and other (19%).

### 3.2. Veterinary Care Staff Survey

In total 173 persons responded to the survey to veterinary care staff, and the median time to finish the survey was 3 min (range < 1–35 min). The majority worked at a veterinary hospital (*n* = 142, 79%) versus a veterinary clinic. Almost half of the respondents were veterinary nurses (*n* = 87, 49%), followed by veterinary surgeons (*n* = 49, 27%) and animal caretaker (*n* = 43, 24%). Forty-five respondents stated that they had plenty of experience of dental care in cats, whereas 111 had some experience and 23 had no experience. Only 3 persons responded that the veterinary facility where they worked did not see dental cases. Table 5 presents the veterinary care staff responses and characteristics.

#### Information by Veterinary Care Staff to Cat Owners

One hundred and sixty veterinary clinics and hospital staff reported on which different ways they used to provide information on prophylaxis to cat owners, see Table 6. Most reported one information type (*n* = 104), but 41 reported 2 ways, 14 three ways and 1 reported 4 ways they used to communicate prophylactic dental care in cats. In addition, 14 persons provided free-text responses on type of information, and this included written advice for the cat after dental treatment at the clinic or hospital (*n* = 4 of which 3 had not marked option “leaflet”). One person did not mark any of the options oral, leaflet or website information but stated that they talk to the owner and give written advice for the cat after dental treatment at the clinic or hospital. Two persons stated that they demonstrate how to brush the teeth on the cat. Sixteen respondents provided additional free text details on what type of product or action they recommend as prophylactic dental care. All of these respondents but one had tooth brushing as a recommendation, and the most common additional advice (*n* = 7) was to use oral chlorhexidine gel if the cat had gingivitis.

Table 7 presents the veterinary care staff routines on advice on preventive dental care and clinical signs related to dental disease. A majority of the veterinary care staff (88%) recommended brushing teeth.

In total, 112 respondents (112/179 = 63%) provided free text answers on what information they give the cat owners regarding common clinical signs for dental problems. The text information was categorised into 12 categories and each respondent could have more than one clinical sign category; modified eating behaviour (*n* = 90, 50%), red and swollen gingiva (*n* = 47, 26%), halitosis (*n* = 40, 22%), change in eating behavior (*n* = 35, 20%), salivation (*n* = 27, 15%), dental calculus (*n* = 23, 13%), pain (*n* = 18, 10%), hiding signs (*n* = 7, 4%), loose teeth (*n* = 5, 3%), weight loss (*n* = 5, 3%), nasal discharge (*n* = 2, 1%), and internal injuries (*n* = 1, 1%). Each respondent provided answers in 1 (*n* = 18), 2 (*n* = 33), 3 (33), 4 (*n* = 23) or 5 (*n* = 5) categories, respectively. The responses for “hiding signs” had one response stating that the cat most commonly hides signs, whereas the other six responses included additional information in other categories such as modified eating behaviour, red gingiva, as well as recommending that most importantly the teeth should be visually inspected regularly.

When asked about how well the veterinary care staff believe that the owners understand their information on the importance of prophylactic dental care, most (*n* = 129, 72%) stated that the owner seemed to understand to some degree, whereas fewer believed that the owner fully understood (*n* = 17, 10%) or did not understand (*n* = 10, 6%).

## 4. Discussion

This study reports owners’ knowledge and perception, and veterinary care staff’s information strategies regarding dental disease and prevention in cats. To our knowledge, there is no previous peer-reviewed study reporting on home care dental routines or veterinary care staff practices in cats. This study was based on a convenience sample of owners and staff; therefore, generalisation to the wider population of cat owners and veterinary care staff needs to be made with caution. The response rate was approximately equally divided between housecat and purebred cat owners. Housecats, which include cats of any mixed breed or of unknown breed (i.e., not formally registered with a purebred cat association), are popular in Sweden and are often insured for veterinary care. For example, housecat is the single most commonly insured cat breed in a major pet insurance provider [17]. Overall, the knowledge on prevention was considered inadequate for ensuring adequate oral health in cats, as owner knowledge is key to regular dental prevention actions. For example, less than half of the owners reported knowledge of preventive measures. This proportion is higher than previously reported from dog owners [18] and is likely an overestimation of the true proportion in cat owners in Sweden, as participation in this cat owner survey likely attracted owners interested in and knowledgeable about dental care in cats.

Oral prophylaxis is the foundation of oral health, and daily plaque removal is considered a necessity for oral health in humans [13]. The requirements for maintaining a dental plaque-free status are similar in cats. In fact, daily tooth brushing was stated as the gold standard and most effective way to remove plaque and maintain gingiva health in cats [19,20]. Even so, we report that only 113 of 405 cat owners (28%) apply tooth brushing as a preventive measure in their cats, and with a low frequency of a daily or every second day routine (*n* = 29 of 113). This is a higher proportion than previously reported among dog owners in Sweden [18], but the proportion is too low for the successful prevention of dental disease in cats. We noted a higher frequency of tooth brushing reported by purebred cat owners; this was not statistically significant but likely due to lack of statistical power. However, more research should be carried out on the efficacy and acceptance of dental foods and dental treats as well as offering these as valid options when brushing teeth is not a practical option or for cats who may not tolerate tooth brushing.

The critical role of client education and effective, preventive oral healthcare has previously been emphasised [9]. A challenge to support good oral health in cats is that oral problems are often not the primary reason for a clinic or hospital visit as the clinical signs of oral problems may often be absent [21]. We report that a majority of owners had knowledge on signs for dental disease, and purebred cat owners had a significantly higher knowledge compared to housecat owners. It can be speculated if purebred cat owners are more interested and informed about preventive dental care measures and signs of dental disease, or if the study design was more likely to attract cat owners who are interested in preventive care routine and more knowledgeable about dental disease. More known factors about the owners are needed to disentangle the potential causal relationship and any confounding variables between purebred or housecat ownership and knowledge on dental disease and prevention, as ownership of purebred cat likely is a marker for unknown factors affecting the knowledge level.

The evidence for chronic discomfort related to dental disease supports an aggressive prophylactic approach to prevent the development and progression of dental disease [21] to reduce pain and increase welfare. Owner compliance is a cornerstone for home care prevention for dental disease [19] and our study indicates that there is an improvement to be made regarding educating and informing cat owners on the necessity of them performing daily dental routines in their homes. Emphasis on annual dental check-ups at the veterinary clinic or hospital should be made by veterinary care staff to improve compliance, as professional oral hygiene recommendations/instructions were reported to improve motivation and compliance [19]. For cats, some individuals may not accept toothbrushing easily and training from a young age is recommended to increase the likelihood of acceptance. In addition, as mentioned above, dental food and dental treats may be recommended as alternatives when tooth brushing is not a practical option. Apart from using toothbrushes suitable for cats size wise, microfiber pull-on fingertip cleaning tools can be used, particularly during the acclimatising period. Barriers to overcome were presented and include the perception that care and prevention are unnecessary, inadequate understanding by pet owners of the need for routine examination of their pets, the cost of professional care, the difficulty of home care, a fear of anesthesia, transporting cats to the veterinary clinic or hospital and resistance to examination or treatment [22,23]. Additionally, owners who understand that preventive care will likely lead to prolonged longevity for the cat would potentially be much more likely to become regular users of veterinary medical services [22]. Education of cat owners and initiation of discussions about periodontal disease and prevention should start when the kitten is young [24].

In this study, 87% of the veterinary care staff report that they always or often inform the cat owners of prophylactic care and dental disease which is comparable to reported findings in dogs [18], thus suggesting similar approaches towards cat and dog owners on this matter. There is however potential for improvement still as 11 % of veterinary care staff do not inform owners of prophylactic care and dental disease. Veterinary clinic staff reported more options on when they provide information to owners compared to veterinary hospital staff, and there was a tendency of clinics having a higher proportion for most occasions when advice was given such as at health visits and if the cat is showing obvious signs of dental disease compared to hospitals. It can only be speculated why clinics had a higher frequency of providing advice regarding dental problems and prevention. For example, clinics might perform more preventive and general health work as a first opinion veterinary care point of contact, as owners may prefer to seek veterinary advice at a smaller, more familiar facility for planned and minor issues. Hospitals have commonly more advanced equipment and educated staff to provide the owners and animals with around-the-clock services also for more advanced health problems. In Sweden, the cost of visiting a hospital is therefore often higher compared to a clinic for similar clinical examinations or treatments as the cost of maintaining the infrastructure and competence, in general, is much higher at the hospital.

In addition to daily tooth brushing, daily additions of dental chews were reported to support oral health and reduce gingivitis and plaque in cats [19]. In the present study, only 14% of the cat owners report that they apply dental chews as a preventive measure but a higher proportion of staff claim to recommend dental treats or other dental products (i.e., not dental food or dental toys). In cats with a history of developing plaque, weekly application of a barrier gel dental product may reduce plaque deposition [20], however, this routine did not affect gingivitis, gingival bleeding, or calculus. Dental chews, food and treats can be regarded as a valuable alternative to tooth brushing, albeit not as effective, as not all owners will be able to handle their cats and comply with tooth brushing recommendations [25].

One key component in increasing the knowledge on and performance of dental preventive measures in cats is to increase the understanding in owners of the need for routine examination of their cats. A rather low proportion of cat owners perceived the information received on dental problems by veterinary care staff as good or very good (31%). In contrast, veterinary care staff reported that they thought owners fully (10%) or partially (72%) understood the information given. In this study, the owners and veterinary care staff were not paired or connected in any way, which reduces the possibility to fully compare the experience of the owners and staff, respectively. However, these perceptions deem further investigation to calibrate the information transmission to increase the likelihood of understanding and satisfaction. We noted that owners who graded the information from their veterinary care staff as “very good” or “good” were more likely to perform preventive measures at home. Suggested improvements are effective communication regarding preventive care and annual (or more often for some individuals) health check-ups. The value of preventive care and early intervention is well-known in human medicine and dentistry, but less so to pet owners [23]. It has been suggested that lack of compliance can be solved by pet owner education, a key benefit of regular veterinary care visits [23]. However, pet owner education is, albeit important, likely at best a potential and partial solution as owners also need to establish routines and habits that they can uphold with adequate regularity. Veterinary clinics and hospitals can provide adequate communication training and support for their staff to successfully inform the owners on the need for regular visits and education.

This study presents that veterinary care staff are regularly providing support and education to cat owners, although the perception of the quality of the information received can be improved. Efforts should be directed towards encouraging cat owners to ensure their cat receives regular health check-ups, even though their cat may not present any clinical signs of disease. At the clinic or hospital, routines and competence in communication should be assessed and developed to ensure that owners receive and understand information on prevention and home care relevant to dental disease.

## 5. Conclusions

In conclusion, the results in the present study show that cat owners have relevant knowledge on dental disease signs and diseases in cats, but the performed preventive measures are inadequate to support good oral health in cats. For example, less than half of the cat owners performed any preventive measures, and 7% of owners reported to perform daily or every second day tooth brushing. Veterinary care staff generally inform cat owners on dental disease and prevention but some owners do not receive information. However, there was a discrepancy between the cat owners’ perception of the information received on dental problems by veterinary care staff and the veterinary care staff’s perception of the degree of understanding regarding the information provided. There is room to develop preventive and communication strategies to increase welfare in cats.

## Figures and Tables

**Table 1:** Description of cat owners’ responses and characteristics from a survey to cat owners on knowledge of dental problems and prophylaxis. Comparison was made between housecat and purebred cat owners. For variables with >2 categories, the statistical test applies to the overall variable, not individual comparisons between categories. * indicates statistical significance.

Variable	All, *n* = 407	Housecats, *n* = 228	Purebred Cats, *n* = 179	Comparison
Indoor		134 (59%)	162 (91%)	*p* < 0.001 *
Age category of cat				*p* = 0.03 *
<1 year	39 (10%)	25 (11%)	14 (8%)	
1–5 years	158 (39%)	73 (32%)	85 (47%)	
5–10 years	123 (30%)	78 (34%)	45 (25%)	
10–15 years	73 (18%)	43 (19%)	30 (17%)	
>15 years	14 (3%)	9 (4%)	5 (3%)	
Previous treatment for dental problems				*p* = 0.53
Yes	112 (28%)	61 (27%)	51 (28%)	
No	290 (71%)	163 (71%)	127 (71%)	
Do not know	5 (1%)	4 (2%)	1 (1%)	
Knowledge signs for dental problems				*p* = 0.07
No	112 (28%)	71 (31%)	41 (23%)	
Yes	295 (72%)	157 (69%)	138 (77%)	

**Table 2:** Information on cat owners’ knowledge on signs for dental problems, from a survey to cat owners in Sweden regarding knowledge on dental problems and prophylaxis, with open-ended questions. Of the 295 owners who claimed to know about signs, 286 gave examples in one or more of the specified variable categories. For each variable, the number of owners that reported knowledge of this variable is included in “All” and then stratified by type of cat. The *p*-value for statistical significance was adjusted using Bonferroni correction to account for multiple testing: threshold for statistical significance was 0.05/9 = 0.0056. * indicates statistical significance.

Variable	All, *n* = 286	Housecats, *n* = 153	Purebred Cats, *n* = 133	Comparison
Modified eating behaviour	166 (58%)	87 (57%)	79 (59%)	*p* = 0.67
Gingivitis	140 (49%)	63 (41%)	77 (58%)	*p* = 0.005 *
Halitosis	101 (35%)	52 (34%)	49 (37%)	*p* = 0.61
Pain	74 (26%)	46 (30%)	28 (21%)	*p* = 0.08
Dental calculus	61 (21%)	32 (21%)	29 (22%)	*p* = 0.86
Feline resorptive lesions (FORL)	47 (16%)	27 (18%)	20 (15%)	*p* = 0.55
Tooth loss	31 (11%)	16 (10%)	15 (11%)	*p* = 0.82
Sialorrhea	30 (10%)	20 (13%)	10 (8%)	*p* = 0.13
Weight loss	15 (5%)	9 (6%)	6 (5%)	*p* = 0.60

**Table 3:** Description of sources of information regarding dental health, for cat owners and by breed type owned (purebred versus housecat). Comparisons between purebred and housecat owners were performed using Chi-squared test except where any cells were *n* < 15 where Fisher’s exact test was used. The *p*-value for statistical significance was adjusted for the number of tests (*n* = 8) using Bonferroni correction: 0.05/8 = 0.00625, and * indicates statistical significance. For the variable Receiving dental information from veterinary sources, the statistical test applies to the overall variable, not individual comparisons between categories.

Variable	All	Housecats	Purebred Cats	Comparison
Source of information Internet	*n* = 353 241 (68%)	*n* = 193 129 (67%)	*n* = 160 112 (70%)	*p* = 0.53
Veterinary care staff	198 (56%)	108 (56%)	90 (56%)	*p* = 0.96
Books/journals	72 (20%)	39 (20%)	33 (21%)	*p* = 0.92
Friend	71 (20%)	36 (19%)	35 (22%)	*p* = 0.45
Course/education	59 (17%)	28 (15%)	31 (19%)	*p* = 0.22
Breeder	36 (10%)	9 (5%)	27 (17%)	*p* < 0.005 *
Other	26 (7%)	11 (6%)	15 (9%)	*p* = 0.13
Do not remember	5 (1%)	3 (2%)	2 (1%)	*p* = 0.59
Receiving dental information from veterinary sources	*n* = 407	*n* = 228	*n* = 179	*p* = 0.79
Very good or good information	127 (31%)	68 (30%)	59 (33%)	
described as “okay”	117 (29%)	67 (29%)	50 (28%)	
Poor or very poor information	163 (40%)	93 (41%)	70 (39%)	

**Table 4:** Owner reported preventive measures on dental health in their cats. In total, 167 cat owners reported on 271 preventive measures they perform. The *p*-value for statistical significance was adjusted using the Bonferroni correction to avoid incorrect significance testing results due to multiple testing (*p*-value for statistical significance = 0.05/5 = 0.01). * indicates statistical significance.

Preventive Measure	All, *n* = 271	Housecats, *n* = 116	Purebred Cats, *n* = 155	Comparison
Tooth brushing	113 (68%)	52 (23%)	61 (34%)	*p* = 0.012
Dental treats	56 (34%)	23 (10%)	33 (18%)	*p* = 0.015
Dental food	37 (22%)	21 (9%)	16 (9%)	*p* = 0.925
Dental toys	32 (19%)	12 (5%)	20 (11%)	*p* = 0.028
Other products such as nutritional supplements	33 (20%)	8 (4%)	25 (14%)	*p* < 0.001 *

**Table 5:** Description of the respondents among veterinary care staff, *n* = 179, from a survey to veterinary clinics and hospitals on information from staff to cat owners regarding dental problems and prophylaxis.

Variable	Category	*n* = 179
Type of veterinary care facility	Clinic	37 (21%)
	Hospital	142 (79%)
Profession	Veterinary surgeon	49 (27%)
	Veterinary nurse	87 (49%)
	Animal caretaker	43 (24%)
Years of experience	0–1 year	14 (8%)
	1–5 years	46 (26%)
	5–10 years	37 (21%)
	10–20 years	56 (31%)
	20–30 years	17 (10%)
	+ 30 years	9 (5%)
Experience dental care in cats	Yes, plenty	45 (25%)
	Yes, some	111 (62%)
	No, none	23 (13%)
If the facility where they work takes dental patients	Yes, equipment available for minor and advanced procedures	166 (93%)
	Yes, equipment available for minor procedures	8 (4%)
	Yes, equipment available for basic cases	2 (1%)
	No, do not take dental cases	3 (1%)
Inform owners of prophylactic care and dental disease	Yes	84 (47%)
	Sometimes	76 (42%)
	No	19 (11%)

**Table 6:** Veterinary care staff responses on what type of information they provide to their cat-owning clients when this information is given and the frequency of this routine. The *p*-value for statistical significance was adjusted for variables where several answers were allowed and several tests performed, using the Bonferroni correction to avoid incorrect significance testing results due to multiple testing (one correction per question: *p*-value for statistical significance = 0.05/4 = 0.013 and 0.05/5 = 0.01, respectively). * indicates statistically significant difference between type of veterinary facility (clinic/hospital).

Variable	All Staff	Clinics	Hospitals	Comparison
Type of information	*n* = 160	*n* = 35	*n* = 125	
Short oral information	109 (68%)	21 (60%)	88 (70%)	*p* = 0.56
Through oral information	62 (39%)	16 (46%)	46 (37%)	*p* = 0.22
Leaflet	36 (23%)	15 (43%)	21 (17%)	*p* = 0.001 *
Websites	11 (7%)	4 (11%)	7 (6%)	*p* = 0.19
When do you give information on dental care?	*n* = 179	*n* = 37	*n* = 142	
Dental related visits	114 (64%)	30 (81%)	84 (59%)	*p* = 0.014
Health visits	108 (60%)	27 (73%)	81 (57%)	*p* = 0.078
After stationary care	30 (17%)	4 (11%)	26 (18%)	*p* = 0.28
If the cat is showing obvious dental problems	123 (69%)	30 (81%)	93 (65%)	*p* = 0.069
Wellness visits/health check ups	94 (53%)	26 (70%)	68 (48%)	*p* = 0.015
How often do you inform cat owners on dental prevention?	*n* = 160	*n* = 35	*n* = 125	*p* < 0.001 *
Daily	32 (20%)	17 (49%)	15 (12%)	
3–7 times per week	30 (19%)	6 (17%)	24 (19%)	
1–3 times per week	44 (28%)	6 (17%)	38 (30%)	
More seldom	54 (34%)	6 (17%)	48 (38%)	

**Table 7:** Veterinary care staff responses regarding type of prophylactic care advice and information on signs of dental disease in cats to cat owners. The *p*-value for statistical significance was adjusted using the Bonferroni correction to avoid incorrect significance testing results due to multiple testing (*p*-value for statistical significance = 0.05/5 = 0.01 and 0.05/6 = 0.008, respectively). * indicates statistically significant difference between types of veterinary facility (clinic/hospital).

Variable	All Staff	Clinics	Hospitals	Comparison
Do you recommend the following prophylactic dental care?	*n* = 160	*n* = 35	*n* = 125	
Tooth brush	157 (88%)	35 (95%)	122 (86%)	*p* = 0.15
Dental food	39 (22%)	12 (32%)	27 (19%)	*p* = 0.08
Other dental products	37 (21%)	13 (35%)	24 (17%)	*p* = 0.015
Dental treats	11 (6%)	1 (3%)	10 (7%)	*p* = 0.33
Dental toy	9 (5%)	2 (5%)	7 (5%)	*p* = 0.91
Information on dental problems	*n* = 179	*n* = 37	*n* = 142	
Dental calculus	154 (86%)	35 (95%)	119 (84%)	*p* = 0.09
Feline resorptive lesions (FORL)	141 (79%)	35 (95%)	106 (75%)	*p* = 0.0082
Gingivitis	113 (63%)	27 (73%)	86 (61%)	*p* = 0.163
Tooth loss	91 (51%)	26 (70%)	65 (46%)	*p* = 0.0079 *
Tooth fracture	63 (35%)	24 (65%)	39 (27%)	*p* < 0.001 *
Malocclusion	37 (21%)	17 (46%)	20 (14%)	*p* < 0.001 *

## Data Availability

Data are available upon request to the senior author. The two surveys, in Swedish, are available upon request to the senior author.
